# The effect of statins on testosterone in men and women, a systematic review and meta-analysis of randomized controlled trials

**DOI:** 10.1186/1741-7015-11-57

**Published:** 2013-02-28

**Authors:** C Mary Schooling, Shiu Lun Au Yeung, Guy Freeman, Benjamin J Cowling

**Affiliations:** 1 CUNY School of Public Health at Hunter College, 2180 Third Avenue, New York, NY 10035, USA; 2 Department of Community Medicine, School of Public Health, Li Ka Shing Faculty of Medicine, The University of Hong Kong, Units 624-627, Core F, Cyberport 3, 100 Cyberport Road, Hong Kong, Hong Kong SAR, China

**Keywords:** androgen, cardiovascular, cholesterol, diabetes, inflammation, statins, testosterone

## Abstract

**Background:**

Statins are extensively used for cardiovascular disease prevention. Statins reduce mortality rates more than other lipid-modulating drugs, although evidence from randomized controlled trials also suggests that statins unexpectedly increase the risk of diabetes and improve immune function. Physiologically, statins would be expected to lower androgens because statins inhibit production of the substrate for the local synthesis of androgens and statins' pleiotropic effects are somewhat similar to the physiological effects of lowering testosterone, so we hypothesized that statins lower testosterone.

**Methods:**

A meta-analysis of placebo-controlled randomized trials of statins to test the *a priori *hypothesis that statins lower testosterone. We searched the PubMed, Medline and ISI Web of Science databases until the end of 2011, using '(Testosterone OR androgen) AND (CS-514 OR statin OR simvastatin OR atorvastatin OR fluvastatin OR lovastatin OR rosuvastatin OR pravastatin)' restricted to randomized controlled trials in English, supplemented by a bibliographic search. We included studies with durations of 2+ weeks reporting changes in testosterone. Two reviewers independently searched, selected and assessed study quality. Two statisticians independently abstracted and analyzed data, using random or fixed effects models, as appropriate, with inverse variance weighting.

**Results:**

Of the 29 studies identified 11 were eligible. In 5 homogenous trials of 501 men, mainly middle aged with hypercholesterolemia, statins lowered testosterone by -0.66 nmol/l (95% confidence interval (CI) -0.14 to -1.18). In 6 heterogeneous trials of 368 young women with polycystic ovary syndrome, statins lowered testosterone by -0.40 nmol/l (95% CI -0.05 to -0.75). Overall statins lowered testosterone by -0.44 nmol/l (95% CI -0.75 to -0.13).

**Conclusions:**

Statins may partially operate by lowering testosterone. Whether this is a detrimental side effect or mode of action warrants investigation given the potential implications for drug development and prevention of non-communicable chronic diseases.

See commentary article here http://www.biomedcentral.com/1741-7015/11/58

## Background

Statins are extensively used in the West for secondary prevention of cardiovascular diseases, and have contributed to a reduction in cardiovascular disease mortality rates. Statin use will become more common globally with the emerging epidemic of cardiovascular disease in developing countries and the increasing availability of statins off patent. Randomized controlled trials (RCTs) show statins reduce mortality rates more than other commonly used lipid-modulating therapies [1,2], such as niacin, fibrates and ezetimibe (where the evidence is limited), and are beneficial for some cardiovascular diseases poorly correlated with cholesterol [3], although statins increase the risk of diabetes [4], with a dose-dependent effect [5]. In RCTs statins also reduce inflammation [6,7] and improve immune function [8,9]. Currently, there is no known, coherent explanation for this specific pattern of pleiotropic effects, and no identified 'active ingredient', despite relevance to drug discovery, existing therapies and modifiable risk factors.

Statins inhibit the enzyme 3-hydroxy-3-methyl-glutaryl-CoA (HMG-CoA) reductase, which reduces cholesterol production. In contrast niacin blocks the breakdown of fats, fibrates activate peroxisome proliferator-activated receptor (PPAR)-α and ezetimibe inhibits cholesterol absorption. All modulate circulating lipids, but only statins also inhibit *de novo *synthesis of cholesterol throughout the body [10]. Concern has always existed that statins might impair testosterone production [11] either by reducing availability of its preferred substrate, that is, locally produced *de novo *cholesterol in the gonads [12] and elsewhere, or by inhibiting steps in the steroidogenesis process [13], but this concern has been considered of little clinical significance [14-16]. Lowering androgens potentially explains some of statins' effects. An RCT among men found testosterone therapy enhanced glucose metabolism [17], whilst impaired glucose metabolism may be a side effect of androgen deprivation therapy [18]. Men with naturally low androgens due to Klinefelter's syndrome are more prone to diabetes [19]. Extensive experimental evidence indicates that testosterone is immunomodulatory [20,21] and impairs immune response [22,23]. Given statins' physiological mechanism would be expected to reduce androgens as well as the similarity between the effects of statins and of reducing androgens, we hypothesized *a priori *that the pleiotropic effects of statins could be due to lowering of androgens, that is, that statins lower androgens and that lower androgens might mediate the pleiotropic effects of statins. Here, we carried out a meta-analysis of placebo-controlled randomized trials, to avoid bias by indication, in men and women to examine whether statins reduced total testosterone.

## Methods

We implemented this meta-analysis following the Preferred Reporting Items for Systematic Reviews and Meta-Analyses (PRISMA) checklist (Additional file 1). Two reviewers (CMS and SLAY) independently searched for and selected studies, resolving differences by consensus. Two statisticians (GF and BJC) extracted information from the selected studies.

### Data sources and searches

We systematically searched the PubMed, Medline and ISI Web of Science databases until the end of 2011 using search term '(Testosterone OR androgen) AND (CS-514 OR statin OR simvastatin OR atorvastatin OR fluvastatin OR lovastatin OR rosuvastatin OR pravastatin)' with the search limited to RCTs of studies in humans in English, because an initial search suggested the relevant literature was in English. From our search, we discarded any studies that were not relevant from the title or abstract and read the remaining to identify placebo-controlled randomized trials of statins with testosterone reported. We also used the references of the selected trials to identify additional relevant trials.

### Study selection

We included any published placebo-controlled randomized trial in English of at least 2 weeks' duration examining the effect of statins on testosterone in adults, because statins act rapidly on cholesterol [11]. We did not consider trials in children, because children have different levels of testosterone, and statins are rarely used in children. We did not otherwise select by participant characteristics, because statins are used for the same purpose in a wide variety of patients, and there is no reason to think that statins have different effects by patient subgroup.

### Data extraction and quality assessment

A statistician (GF) extracted information by trial arm on number of participants, testosterone assays used, and mean and standard deviation of the change in testosterone during the trial. Where this information was not provided in full, the statistician (GF) made conservative estimates based on the information available. A second statistician (BJC) then checked the information extracted and any estimates. The reviewers (CMS and SLAY) independently used an established tool to evaluate the quality of each trial [24], and a sensitivity analysis was done excluding the lower quality trials.

### Data synthesis and analysis

We used funnel plots to assess publication bias, and I^2 ^to assess heterogeneity between trials. To obtain an estimate of the difference in mean testosterone between statin and placebo groups, we combined the results of the selected trials using inverse variance weighting and a fixed or random effects model depending on the level of heterogeneity, using the 'metacont' function from the 'meta' package of R 2.14.1 (R Development Core Team, Vienna, Austria). We considered men and women together and separately because of the different hormone milieu by sex. We did no other subgroup analysis.

This study is an analysis of published data, which does not require ethics committee approval.

## Results

**Figure 1:**
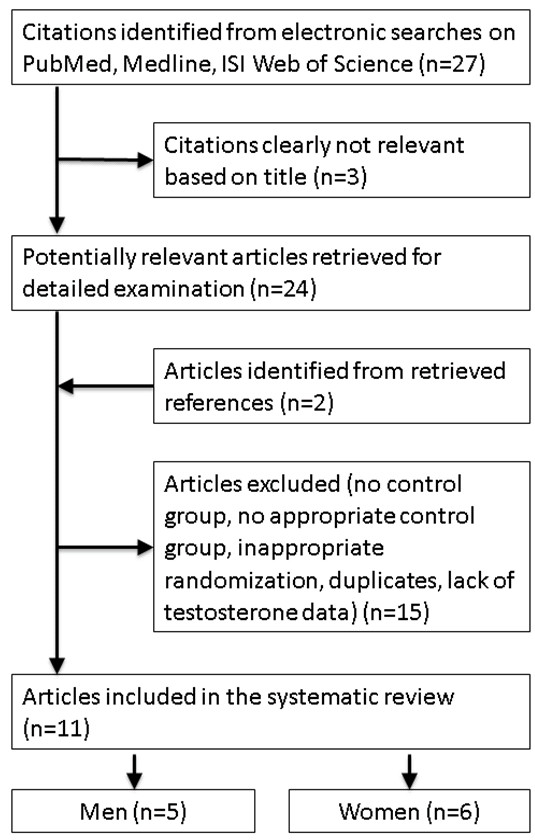
**Selection process for the placebo-controlled randomized trials of the effects of statins on testosterone**.

The initial searches yielded 27 studies. Two additional trials [11,25] were found from the bibliographic search. We discarded 18 studies. Three were clearly not relevant based on title, four had no control group, three had the control group taking other drugs (namely neomycin, cholestyramine or other lipid-lowering drugs and atorvastatin plus ezetimibe), four were not randomized controlled trials of statins, two were largely duplicates of other included publications [26,27] and two provided insufficient information. Details of these 18 excluded studies are given in Additional file 2. Of the 11 trials remaining, 1 small trial of 15 men and 7 women providing only graphs had an implausibly high post-intervention value of testosterone for women in the placebo group (mean approximately 5.2 nmol/l for 3 women, no standard deviation given) [28], so these 7 women were also excluded. Figure 1 shows the search strategy resulting in these 11 placebo-controlled randomized trials.

Table 1 shows there were 5 trials over 25 years of 501 men, mainly middle aged with hypercholesterolemia, taking typical doses of statins. There were 6 recent trials of 368 young women with polycystic ovary syndrome. Most of the trials were in western settings and were carried out by teams affiliated with or funded by pharmaceutical companies. Most of the trials were of average quality as shown in Additional file 3, with the larger, more recent ones generally of higher quality.

**Table 1:** Characteristics of placebo-controlled randomized trials giving the effects of statins on testosterone

Lead author/publicationyear/reference	Study					Participants				Authors: funding and affiliations
		
	Setting	Duration	Statin and daily dose	Testosterone assessment method	Comments	Men	Women	Mean age, years	Health status	
									
						Statin/placebo (no. completed study)				
Tobert 1982 [11]	Europe	4 weeks	Lovastatin, multiple doses	Various radio immunoassays		47/10^a^	0	29	Healthy volunteers	Affiliations include MSD
Dobs 2000 [16]	US	24 weeks	Simvastatin 20/40 mg/pravastatin 40 mg	Radio immunoassay		85/28	0	40	Hypercholesterolemia	Affiliations include Merck
Dobs 2000 [15]	US	12 weeks	Simvastatin 80 mg	Radio immunoassay		37/39	0	45	High LDL cholesterol	Affiliations include Merck
Hyyppä 2003 [29]	Finland	12 weeks	Simvastatin 20 mg	Direct competitive immunoassay		120/120	0	48	Hypercholesterolemia	None given
Boehm 2004 [28]	Germany	3 months	Pravastatin 40 mg	Competitive electrochemiluminescence immunoassay		6/9	4/3^b^	65	Hypercholesterolemia	None given
Banaszewska 2007 [26]	Poland	12 weeks	Simvastatin 20 mg	Specific chemiluminescence assay	Also using OCPs	0	45/48	24	PCOS	NIH and drug donations
Banaszewska 2009 [27]	Poland	3 months	Simvastatin 20 mg	Specific electrochemiluminescence assay	Also using metformin	0	37/36	25	PCOS	NIH and Polish State Committee for Scientific Research
Sathyapalan 2009 [30]	UK	12 weeks	Atorvastatin 20 mg	Chemiluminescent immunoassay		0	19/18	28	PCOS	Pfizer
Kazerooni 2010 [32]	Iran	12 weeks	Simvastatin 20 mg	Radio immunoassay	Also using metformin	0	42/42	25	PCOS	None
Raja-Khan 2011 [31]	US	6 weeks	Atorvastatin 40 mg	Not given		0	9/11^c^	33	PCOS	NIH, Penn State Univeristy, Pfizer
Rashidi 2011 [33]	Iran	8 weeks	Simvastatin 20 mg	Chemiluminescence assay	Also receiving IVF treatment	0	32/29	25	PCOS	Daru Darman Pars Co.

^a^Subjects with missing data excluded, but number excluded not clearly given; at least two from the statin arm were excluded.

^b^Data not used because of an implausible value for testosterone at study end in the placebo group.

^c^Two subjects with missing data excluded, but not clear from which arm(s).

IVF = *in vitro *fertilization; MSD = Merck, Sharp and Dohme; NIH = National Institute of Health; OCP = oral contraceptives; PCOS = polycystic ovary syndrome.

**Figure 2:**
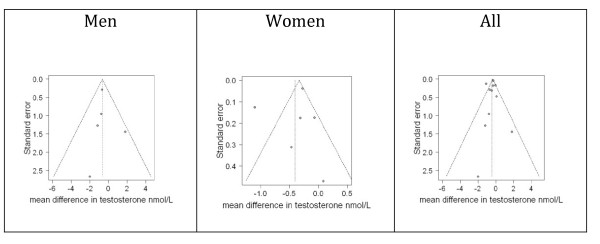
**Funnel plots of placebo-controlled randomized trials examining the effects of statins on testosterone by sex and for men and women together**.

Funnel plots gave little indication of publication bias among men (Figure 2), but among women, the trials were more diverse. For completeness Figure 2 also shows the funnel plot for men and women together.

**Figure 3:**
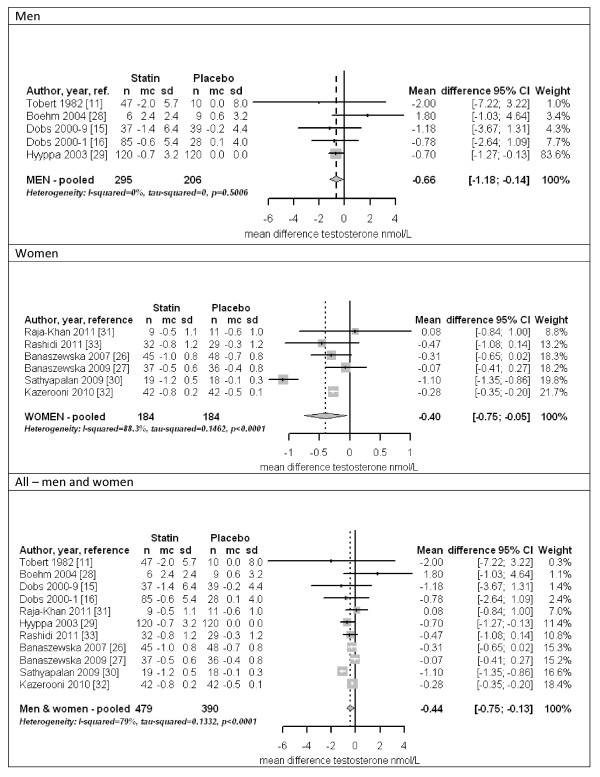
**Forest plots of placebo-controlled randomized trials examining the pooled effects of statins on testosterone for men (top panel), women (middle panel) and both sexes (bottom panel)**. In Kazerooni *et al. *[32], the reported SD was much smaller than in other trials, whereas the reported SD multiplied by the square root of the sample size was similar to the SDs reported in other trials. If we were to assume that the reported SD was actually the standard error, the pooled estimate for women would be -0.40 nmol/l (95% CI -0.83 to 0.03) and the overall pooled estimate would be -0.44 nmol/l (95% CI -0.80 to -0.08). Mean = mean difference; SD = standard deviation.

Figure 3 (top panel) shows a forest plot for men. The trials were homogeneous (I^2 ^= 0%), with the overall estimate within the confidence intervals of all trials. Statins lowered testosterone by about 3.4% among men (-0.66 nmol/l, 95% confidence interval (CI) -0.14 to -1.18) in both fixed and random effects models, and the estimate was similar (-0.73 nmol/l, 95% CI -0.20 to -1.26) including only the higher quality trials [15,16,29]. Figure 3 (middle panel) shows a forest plot for women. The trials were heterogeneous (I^2 ^= 90.1%). Statins lowered testosterone by about 12.3% among women (-0.40 nmol/l, 95% CI -0.05 to -0.75) in a random effects model, with similar results when the most extreme study [30] was omitted. The estimate for women was also similar, but included no effect (-0.50 nmol/l, 95% CI 0.06 to -1.06) when considering only the higher quality trials [30-33]. For completeness, Figure 3 (bottom panel) shows a forest plot for men and women together. The trials were heterogeneous (I^2 ^= 79%). Statins lowered testosterone overall (-0.44 nmol/l, 95% CI -0.13 to -0.75) in a random effects model; results were similar when each of the three most influential trials was removed, or when only considering the higher quality trials (-0.57 nmol/l, 95% CI -0.12 to -1.02) [15,16,29-33].

## Discussion

This meta-analysis of placebo-controlled randomized trials suggests statins reduce testosterone. Among men the evidence was homogeneous and largely related to typical doses for the target group using statins for the prevention of cardiovascular disease, where statins potentially lowering testosterone has always been a concern [11,12,14-16]. Among women less concern has existed [34]. Very few trials have examined testosterone among women using statins for the prevention of cardiovascular disease, and our findings were less robust for women than men. Nevertheless, statins have recently been discovered as an effective anti-androgen treatment for polycystic ovary syndrome [25,35]. A recent meta-analysis of statin therapy for women with polycystic ovary syndrome concluded that statins reduced testosterone based on the same literature [36].

To the best of our knowledge no previous meta-analysis of placebo-controlled randomized trials has assessed the effect of statins on testosterone among men. Two trials comparing simvastatin 80 mg/day with 40 mg/day among in total 640 men found median testosterone lower by 10.3% and 7.5% respectively after 48 weeks [34], consistent with the 3.4% reduction here among men mainly using simvastatin 20 mg/day, suggesting a possible dose response of statins on testosterone.

The clinical significance of this reduction in testosterone with statins is difficult to gauge. The normal range of testosterone is wide [37] and sexual function similar across the range [38]. Erectile dysfunction is a rare side effect of statins [39], perhaps because statins' have beneficial effects on cardiovascular function that would counteract changes of this magnitude in testosterone. However, large changes in testosterone (and libido) can occasionally occur with statin use [40], which are reversible by statin withdrawal [40]. The impact on population health may be more germane, where statins causing diabetes could be another side effect. We are not aware of any study examining whether lowering testosterone mediates the effect of statins on diabetes, as this possibility has not, to the best of our knowledge, been considered before, and remains speculative. Moreover, observational studies suggest serum testosterone has sex-specific physiological effects on diabetes, negative among men [41], but positive among women [41], when statins increase diabetes in both sexes [4,5,42]. We could not identify any RCT confirming testosterone therapy increases diabetes incidence among women. In postmenopausal women, RCTs of testosterone therapy indicate little effect on glucose metabolism [43]. In younger women, some RCTs indicate that low doses of testosterone may improve glucose metabolism [44,45]; female to male transsexuals given high doses of testosterone have improved glucose metabolism [46]. Nevertheless, distinct effects of testosterone on diabetes may occur by dose, sex and age with the reduction in testosterone with statin treatment insufficient to modulate diabetes risk consistently. This meta-analysis also raises the question as to whether lowering testosterone is a side effect of statin therapy or contributes to statins' mode of action, which could inform new treatments and prevention policies. Observationally testosterone is inversely associated with cardiovascular mortality [47]; whether testosterone is causal or a marker of health is unknown [47]. No RCT has shown testosterone therapy reduces cardiovascular events; two RCTs of testosterone therapy were halted because of adverse, mainly cardiovascular events, among men allocated to testosterone [48,49]. Natural experiments suggest lower testosterone protects against specifically ischemic heart disease mortality, with a relatively lower risk in men legally castrated [50] or with Klinefelter's syndrome [51]. Physiologically lowering testosterone may reduce thromboxane and platelet activation [52], specifically relevant to reducing ischemic cardiovascular disease but not to diabetes. However, whether lowering testosterone with statin treatment modulates cardiovascular disease, via these or other pathways, has not been examined.

Despite providing a meta-analysis of all known placebo-controlled randomized trials, limitations exist. First, given when they were conducted, not all the trials had high quality scores. However, they had the expected effects on lipids. Second, not all the trials were intended to assess the effects of statins on testosterone. However, the trial, largely designed for this purpose [29], is influential, and alone found statins reduced testosterone [29]. Third, testosterone was not assayed in the same way in all trials (Table 1). Testosterone is difficult to measure, particularly among women. Heterogeneity for women could be due to differing, possibly suboptimal, testosterone assay techniques, for which we compensated by using a random effects model. Imprecision in the assays also reduces power but would not bias a comparison between statin and placebo groups unless statins interfered with the testosterone assay. Steroids can cause assay interference. We could find no evidence that statins cause assay interference. Fourth, too few trials existed to assess dose-response effects or compare hydrophilic (fluvastatin, rosuvastatin, and pravastatin) to lipophilic (atorvastatin, lovastatin, and simvastatin) statins. However, atorvastatin, pravastatin or simvastatin do not differ substantially in their effects on cardiovascular disease [53]. Fifth, other RCTs may have assessed but not reported the effects of statins on testosterone. Given, the concern that statins lower testosterone [11,12,14-16], these might perhaps be trials where statins reduced testosterone among men. Sixth, RCTs are not always tagged as such and could be missed. To check we searched using 'trial' as a search term instead of restricting the search to RCTs, which gave the same selection (data not shown). Seventh, evidence concerning the effect of statins on testosterone in post-menopausal women is lacking. Eighth, considering the effects of statins on testosterone for men and women together may be invalid, hence results stratified and pooled by sex. Ninth, the effect of statins on testosterone in different ethnic groups is lacking. Effects might differ in settings, such as China, where peak testosterone [37,54], androgen related parameters [55,56] and ischemic heart diseases mortality rates [57] are all lower than in western populations. Finally, cross-sectional studies were not included, because these provide evidence from which it is difficult to assess causality. The larger cross sectional studies only considered men and generally observed lower testosterone among statin users than non-users [58-60].

## Conclusions

This meta-analysis shows that statins reduce testosterone. This finding does not demonstrate that androgens mediate any health effect of statins, but raises the question as to whether testosterone modulation plays a role in statins' effects on health, particularly among men where testosterone is an important hormone. Whether reducing testosterone enhances or impairs the protective effects of statins on cardiovascular mortality needs to be determined urgently, because it could enable the development of enhanced statin based treatments, the development of other drugs in the same class and the identification of potentially modifiable population-wide risk factors for several non-communicable chronic diseases.

## Abbreviations

HMG-CoA: 3-hydroxy-3-methyl-glutaryl-CoA reductase; PPAR: peroxisome proliferator-activated receptor; PRISMA: Preferred Reporting Items for Systematic Reviews and Meta-Analyses; RCT: randomized controlled trial.

## Competing interests

BJC has received research funding from MedImmune Inc., and consults for Crucell MV. The authors report no other potential conflicts of interest.

## Authors' contributions

CMS originated the idea for the paper. CMS and SLAY carried out the systematic search. GF and BJC did the data extraction and analysis. CMS drafted the paper with assistance from SLAY, GF and BJC. All authors reviewed the paper critically and have read and approved the manuscript for publication. All authors had full access to all the data in the study and take responsibility for the integrity of the data and the accuracy of the data analysis. CMS is the guarantor. All authors have read and approved the final manuscript.

## Pre-publication history

The pre-publication history for this paper can be accessed here:

http://www.biomedcentral.com/1741-7015/11/57/prepub

## Supplementary Material

Additional file 1 **Completed Preferred Reporting Items for Systematic Reviews and Meta-Analyses (PRISMA) checklist for this study**. Information as to how this review was conducted. Click here for file

Additional file 2 **Studies excluded after scrutiny with reason for exclusion**. A list, with references, of all the studies excluded from this meta-analysis. Click here for file

Additional file 3 **Quality assessment of the selected placebo controlled randomized controlled trials of the effects of statins on testosterone **[24]. Quality assessment of each trial. Click here for file
