# Supplementary material for: The effect of statins on testosterone in men and women, a systematic review and meta-analysis of randomized controlled trials
Source: BMC Med. 2013 Feb 28;11:57. doi: 10.1186/1741-7015-11-57 (PMC3621815; doi:10.1186/1741-7015-11-57)
Supplement: Additional file 2 — Studies excluded after scrutiny with reason for exclusion. A list, with references, of all the studies excluded from this meta-analysis. [file 1741-7015-11-57-S2.DOCX]

**Additional file 2: Studies excluded after scrutiny with reason for exclusion**

| Reason for exclusion | # | study |
| --- | --- | --- |
| Not relevant from title | 3 | Solomon KR et al. [1], Moyad MA et al. [2], Stephenson AJ et al. [3] |
| No control group | 4 | Kaya C et al. [4], Berberoglu Z et al. [5], Ormiston T et al. [6], Kaya C et al. [7] |
| Control group taking other drugs | 3 | Neomycin in Farnsworth WH et al. [8],  Cholestyramine or other lipid-lowering drugs in Dobs AS et al. [9],  Atorvastatin plus ezetimibe in Kanat M et al. [10] |
| Not randomized on statin treatment | 4 | Sathyapalan T et al. [11], Sathyapalan T el al. [12] (epublished in 2011), Peck A et al. [13], Corona et al. [14] |
| Duplicates of other included studies | 2 | Duleba et al. [15], Banaszewska et al. [16] |
| Insufficient information on testosterone | 2 | Kjaer K et al. [17] did not report testosterone |
|  |  | Jay RH et al. [18] a small trial of 17 men and 6 women from over 20 years ago with no information about placebo group or differences between placebo and statin group |
| Total excluded | 18 |  |

1. Solomon KR, Freeman MR: **The complex interplay between cholesterol and prostate malignancy**. *Urol Clin North Am* 2011, **38:**243-259.

2. Moyad MA: **Promoting general health during androgen deprivation therapy (ADT): a rapid 10-step review for your patients**. *Urol Oncol* 2005, **23:**56-64.

3. Stephenson AJ, Abouassaly R, Klein EA: **Chemoprevention of prostate cancer**. *Urol Clin North Am* 2010, **37:**11-21, Table.

4. Kaya C, Pabuccu R, Cengiz SD, Dunder I: **Comparison of the effects of atorvastatin and simvastatin in women with polycystic ovary syndrome: A prospective, randomized study**. *Exp Clin Endocrinol Diabetes* 2010, **118:**161-166.

5. Berberoglu Z, Guvener N, Asik M, Yazici AC, Bayraktar N: **Effects of Achieving LDL-Cholesterol Levels <70 mg/dL With Simvastatin or Atorvastatin on Steroidogenesis in High-Risk Diabetic Patients** . *Endocrinologist* 2009, **19:**102-107.

6. Ormiston T, Wolkowitz OM, Reus VI, Johnson R, Manfredi F: **Hormonal changes with cholesterol reduction: a double-blind pilot study**. *J Clin Pharm Ther* 2004, **29:**71-73.

7. Kaya C, Cengiz SD, Berker B, Demirtas S, Cesur M, Erdogan G: **Comparative effects of atorvastatin and simvastatin on the plasma total homocysteine levels in women with polycystic ovary syndrome: a prospective randomized study**. *Fertil Steril* 2009, **92:**635-642.

8. Farnsworth WH, Hoeg JM, Maher M, Brittain EH, Sherins RJ, Brewer HB, Jr.: **Testicular function in type II hyperlipoproteinemic patients treated with lovastatin (mevinolin) or neomycin**. *J Clin Endocrinol Metab* 1987, **65:**546-550.

9. Dobs AS, Sarma PS, Schteingart D: **Long-term endocrine function in hypercholesterolemic patients treated with pravastatin, a new 3-hydroxy-3-methylglutaryl coenzyme A reductase inhibitor**. *Metabolism* 1993, **42:**1146-1152.

10. Kanat M, Serin E, Tunckale A, Yildiz O, Sahin S, Bolayirli M, Arinc H, Dirican A, Karagoz Y, Altuntas Y, Celebi H, Oguz A: **A multi-center, open label, crossover designed prospective study evaluating the effects of lipid lowering treatment on steroid synthesis in patients with Type 2 diabetes (MODEST Study)**. *J Endocrinol Invest* 2009, **32:**852-856.

11. Sathyapalan T, Kilpatrick ES, Coady AM, Atkin SL: **Atorvastatin pretreatment augments the effect of metformin in patients with polycystic ovary syndrome (PCOS)**. *Clin Endocrinol (Oxf)* 2010, **72:**566-568.

12. Sathyapalan T, Smith KA, Coady AM, Kilpatrick ES, Atkin SL: **Atorvastatin therapy decreases androstenedione and dehydroepiandrosterone sulphate concentrations in patients with polycystic ovary syndrome: randomized controlled study**. *Ann Clin Biochem* 2012, **49:**80-85.

13. Peck A, Chaikittisilpa S, Mirzaei R, Wang J, Mack WJ, Hodis HN, Stanczyk FZ: **Effect of statins on estrogen and androgen levels in postmenopausal women treated with estradiol**. *Climacteric* 2011, **14:**49-53.

14. Corona G, Boddi V, Balercia G, Rastrelli G, De VG, Sforza A, Forti G, Mannucci E, Maggi M: **The effect of statin therapy on testosterone levels in subjects consulting for erectile dysfunction**. *J Sex Med* 2010, **7:**1547-1556.

15. Duleba AJ, Banaszewska B, Spaczynski RZ, Pawelczyk L: **Simvastatin improves biochemical parameters in women with polycystic ovary syndrome: results of a prospective, randomized trial**. *Fertil Steril* 2006, **85:**996-1001.

16. Banaszewska B, Pawelczyk L, Spaczynski RZ, Duleba AJ: **Effects of simvastatin and metformin on polycystic ovary syndrome after six months of treatment**. *J Clin Endocrinol Metab* 2011, **96:**3493-3501.

17. Kjaer K, Hangaard J, Petersen NE, Hagen C: **Effect of simvastatin in patients with type I (insulin-dependent) diabetes mellitus and hypercholesterolemia**. *Acta Endocrinol (Copenh)* 1992, **126:**229-232.

18. Jay RH, Sturley RH, Stirling C, McGarrigle HH, Katz M, Reckless JP, Betteridge DJ: **Effects of pravastatin and cholestyramine on gonadal and adrenal steroid production in familial hypercholesterolaemia**. *Br J Clin Pharmacol* 1991, **32:**417-422.
