# Supplementary material for: The effect of statins on testosterone in men and women, a systematic review and meta-analysis of randomized controlled trials
Source: BMC Med. 2013 Feb 28;11:57. doi: 10.1186/1741-7015-11-57 (PMC3621815; doi:10.1186/1741-7015-11-57)
Supplement: Additional file 3 — Quality assessment of the selected placebo controlled randomized controlled trials of the effects of statins on testosterone [24]. Quality assessment of each trial. [file 1741-7015-11-57-S3.DOCX]

**Additional file 3: Quality assessment of the selected placebo-controlled randomized trials of the effects of statins on testosterone [24]**

|  | Randomization | Treatment allocation concealed | Group similarity | Eligibility listed | Outcome assessor blinded | Care provider blinded | Subject masked | Point estimates and variability for outcome | ITT | Remarks | Total of yeses |
| --- | --- | --- | --- | --- | --- | --- | --- | --- | --- | --- | --- |
| Tobert 1982 [11] | Yes, but method not specified | Don't know | Don't know | Yes | Yes | n/a | Yes | Yes | No |  | 5 |
| Dobs AS 2000-1 [16] | Yes | Yes | Yes | Yes | Yes | n/a | Yes | Yes | No |  | 7 |
| Dobs AS 2000-9 [15] | Yes, but method not specified | Don't know | Yes | Yes | Yes | n/a | Yes | Yes | No |  | 6 |
| Hyyppä MT, 2003 [29] | Yes, but method not specified | Don't know | Yes | Yes | Yes | n/a | Yes | Yes | Yes |  | 7 |
| Böhm 2004 [28] | Yes, but method not specified | Don't know | Yes | No | Yes | n/a | Yes | No | Yes |  | 5 |
| Banaszewska B 2007 [26] | Yes | Yes | Yes | Yes | No | n/a | No | Yes | No |  | 5 |
| Banaszewska B 2009 [27] | Yes | Yes | Yes | Yes | No | n/a | No | Yes | No |  | 5 |
| Sathyapalan T 2009 [30] | Yes | Yes | Yes | Yes | Yes | n/a | Yes | Yes | No |  | 7 |
| Kazerooni T 2010 [32] | Yes, but method possibly ‘insecure’ | Yes | Yes | Yes | Yes | n/a | Yes | Yes | Yes |  | 8 |
| Raja-Khan 2011 [31] | Yes | Yes | Yes | Yes | Yes | n/a | Yes | Yes | Yes | Stopped early after running out of money | 8 |
| Rashidi B 2011 [33] | Yes | Yes | Yes | Yes | Yes | n/a | Yes | Yes | No |  | 7 |
